# Enabling 3D-Liver Perfusion Mapping from MR-DCE Imaging Using Distributed Computing

**DOI:** 10.1155/2013/471682

**Published:** 2013-02-26

**Authors:** Benjamin Leporq, Sorina Camarasu-Pop, Eduardo E. Davila-Serrano, Frank Pilleul, Olivier Beuf

**Affiliations:** ^1^Université de Lyon, CREATIS, CNRS UMR 5220, Inserm U1044, INSA-Lyon, Université Lyon 1, 69622 Villeurbanne Cedex, France; ^2^Departement d'imagerie Digestive, Hospices Civils de Lyon, CHU Edouard Herriot, 69008 Lyon, France

## Abstract

An MR acquisition protocol and a processing method using distributed computing on the European Grid Infrastructure (EGI) to allow 3D liver perfusion parametric mapping after Magnetic Resonance Dynamic Contrast Enhanced (MR-DCE) imaging are presented. Seven patients (one healthy control and six with chronic liver diseases) were prospectively enrolled after liver biopsy. MR-dynamic acquisition was continuously performed in free-breathing during two minutes after simultaneous intravascular contrast agent (MS-325 blood pool agent) injection. Hepatic capillary system was modeled by a 3-parameters one-compartment pharmacokinetic model. The processing step was parallelized and executed on the EGI. It was modeled and implemented as a grid workflow using the Gwendia language and the MOTEUR workflow engine. Results showed good reproducibility in repeated processing on the grid. The results obtained from the grid were well correlated with ROI-based reference method ran locally on a personal computer. The speed-up range was 71 to 242 with an average value of 126. In conclusion, distributed computing applied to perfusion mapping brings significant speed-up to quantification step to be used for further clinical studies in a research context. Accuracy would be improved with higher image SNR accessible on the latest 3T MR systems available today.

## 1. Introduction

Liver fibrosis is an important cause of mortality and morbidity and contributes substantially to increase health care costs in patient with chronic liver diseases [1]. Fibrosis can lead to cirrhosis, for which the complications such as hepatic decompensation, hepatocellular carcinoma, and portal hypertension involve growing public health concerns. Cirrhosis and chronic liver disease were the 10th leading cause of death for men and the 12th for women in the United States in 2001, leading to the death of about 27,000 people each year [2]. Cirrhosis was first considered as an irreversible process, but, with the growing understanding of hepatic fibrogenesis mechanisms, more effective treatments have been developed [3, 4]. However, the latter must be initiated at a specific and early stage in fibrous development, and their administration requires regular clinical followup. While histological analysis after liver biopsy is the gold standard for the diagnosis, inherent risk of a recognized morbidity and mortality renders this method unsuitable for clinical monitoring [5, 6]. Furthermore liver biopsies have other limitations such as interobserver variability and sampling errors [7]. It has been demonstrated that perfusion imaging has the potential to detect and assess vascular modifications [8] associated with liver fibrosis [9]. Several studies, using magnetic resonance dynamic contrast-enhanced imaging (MR-DCE) to quantify liver perfusion, have shown that some perfusion parameters were relevant indicators for liver fibrosis assessment [10–12]. In a previous work, an MRI protocol associated to a dedicated processing step to quantify liver perfusion was developed [12, 13]. Several parameters showed significant correlations between hepatic perfusion modifications and fibrosis stage. Results demonstrated that MR perfusion imaging could be a noninvasive method for the clinical followup in patient with chronic liver diseases. Nevertheless, the evaluation was restricted to an ROI, and regional variations often met in diffuse liver diseases could not be observed. ROI-based perfusion quantification already requires heavy processing methods such as image registration, denoising, and data fitting. Processing time drastically increases and becomes really prohibitive for clinical application for 2D or 3D mapping. In this context, parallel computing on distributed infrastructures such as clusters, grids, or clouds proves to be an interesting solution. Such infrastructures can bring significant speedup for a large spectrum of applications from various scientific domains. They have already been used for medical imaging as described in [14, 15] but never before for 3D-liver perfusion mapping. Significant effort has been put in rendering distributed infrastructures as user friendly as possible. Nevertheless, new applications still require extra work for adapting (porting) them on the considered infrastructure. This work describes an MR acquisition protocol and a processing method using distributed computing on the European Grid Infrastructure (EGI) to allow 3D liver perfusion parametric mapping after MR-DCE imaging with the MS-325 blood pool agent. Processing speed, reproducibility, and accuracy were assessed and adequate acquisition requirements were defined.

## 2. Materials and Methods

### 2.1. Subjects

The study protocol was approved by the local experimentation ethics committee, and informed consent was obtained from each patient. Privacy rights of subjects have always been observed. Seven subjects (4 women, 3 men; average age, 40 ± 12 years; mean weight, 75 ±  8 kg) were enrolled. Among this group, one healthy subject was used as control and six patients with chronic liver diseases were prospectively enrolled (maximum prospective period of one month) after having had a liver biopsy. Biopsies were performed by percutaneous sampling of the right lobe with a 15-gauge needle. All biopsies were 1.5 cm or more in length. Tissue samples were fixed in buffered formalin and embedded in paraffin. 4 µm-thick sections were stained with hematoxylin-eosin-saffron, iron stain, and Masson trichrome reagents and evaluated by two pathologists. The histopathological evaluation was performed masked from any clinical information. Fibrosis was evaluated on trichrome-stained slides according to the METAVIR classification [16] (*score F0*: absence of fibrosis; *score F1*: portal fibrosis; *score F2*: portal fibrosis with isolated bridges *score F3*: fibrosis with numerous bridges without cirrhosis; *score F4*: cirrhosis).

### 2.2. 3D MR Dynamic Acquisition

Acquisitions were performed using a Siemens Magnetom Symphony Maestro Class 1.5T imaging system (Siemens Medical Solutions, Erlangen, Germany). A T_1_-weighted VIBE 3D sequence with a parallel imaging technique was used (GRAPPA, *R*-factor = 2). The sequence parameters were as follows: TE/TR/*α*, 1.22/2.87 ms/12°; K-space partial filling, 6/8th according to slice and phase direction; reduction of the slice and phase encoding step, 63 and 50%, respectively. The plane was coronal oblique with a rectangular FOV (400 × 300 mm^2^) for a rebuild matrix of 256 × 192 pixels with right/left phase-encoding direction. The rationale behind the use of coronal imaging was to minimize the flow-related enhancement of the aorta. Moreover, it allowed covering a larger liver volume. Volume angulation was not systematic and aorta orientation independent. The exploratory volume was acquired with a 1-sec temporal resolution with 6.4 cm slab thickness (16 slices of 4 mm). The signal was collected using two circularly polarized phased array coils (CP Body Array and CP Spine Array) with a bandwidth of 650 Hz·pixel^−1^. Acquisition has begun at the time of injection of the contrast medium (MS-325;Epix Pharmaceutical, Inc., Lexington, MA, USA), and it continued for 2 minutes [12, 13]. Patients were instructed to breathe calmly. All subjects were asked to undergo fast before MR acquisition. Injection was performed with an injection rate of 1 mL·s^−1^, a posology of 0.03 mM·Kg^−1^, and flushed with 25 mL of physiologic saline injected at the same rate. Finally, sixteen 2D + *t* volumes with *t* = 120 were acquired leading to 1920 images per examination.

### 2.3. Images Preprocessing

Images were first imported on a personal computer running an in-house developed application written in Matlab r2010a (The MathWorks, Natick, MA, USA).

Due to free-breathing acquisition, spatial shifts linked to motion had to be corrected. Hence, each volume was automatically registered. The registration method ignored nonrigid aspects of liver transformation during breathing. Only translations and rotations (rigid transformations) were taken into account. This method consisted in the estimation of the transformation vector needed to register each moving images in relation to a static reference image. For each image from 2D  +  *t*  volumes, a pixel-based method was used (iconic approach) to control the transformation of an input image. An error measure was used to measure the registration error between the moving and static image. The reduced-memory Broyden-Fletcher-Goldfarb-Shanno (BFGS) quasi-Newton algorithm was used to move the control points to achieve affine rigid registration between both images with a minimal registration error. Then pixels were interpolated with a bicubic method.

Secondly, native 2D +   *t* volumes were converted in to 2D + *t* MS-325 mass concentration maps from a pixel-by-pixel operation based on the relationship between signal intensity and MS-325 concentration. The latter was established in a previous work using a calibration phantom [13].

Thirdly, native arterial and portal input functions, *C*_*A*_(*t*) and *C*_*P*_(*t*), were measured using squared ROIs of 25 pixels placed by an experienced radiologist (*F*.P. 12 years of postgraduate experience in digestive imaging) at the level of the abdominal aorta close to the cœliac trunk and the main portal vein. Finally, definitive arterial and portal input functions were converted into continuous form (function of the time) instead of vectorial form (discrete form), by an interpolation using spline curves. Measurements were previously filtered using a moving average filter to reduce noise effect.

### 2.4. Image Modeling

Hepatic capillary system was modeled by a 3-parameter one-compartment pharmacokinetic model adapted to hepatic dual supply (portal and arterial). The central compartment includes the hepatic sinusoids and the space of Disse. The leakage of tracer through venous washout is carried out exponentially over time and the inverse of the constant of elimination is the mean transit time (MTT). The equation describing this model is as follows:

(1) C(t)=ρ[CA(t−τA)×ϕA+Cp(t−τP)×ϕP]⊗e−t/MTT,

where ⊗  designates the convolution product and *ρ* the volemic mass considered to be equal to 1 g·mL^−1^. The parameters,  *ϕ*_*A*_ and *ϕ*_*P*_, are the arterial and portal perfusion, respectively, expressed as mL·100 g^−1^·min^−1^. *C*_*A*_ and *C*_*P*_, are respectively, the arterial and portal input functions. The two delays, *τ*_*A*_ and *τ*_*P*_, take into account the temporal offset between central compartment input and measured input from arterial and portal ROIs. While   *ϕ*_*A*_,  *ϕ*_*P*_, and MTT are model parameters, delays are independent of the fit procedure. The hepatic perfusion index (HPI), defined as the arterial perfusion to total perfusion (arterial + portal perfusion) ratio, was also calculated. For each part of the image, pixel-by-pixel tissular time activity curves were obtained and a nonlinear least-square fit was performed according to the model previously described(1) using the Levenberg-Marquard algorithm. Because some coefficients are closely connected, in particular portal perfusion and arterial perfusion with arterial and portal delay, the results of optimization were strongly influenced by the choice of starting coefficients, and algorithm may converge to local minima. In order to improve the robustness and reliability of optimization, but also to avoid any convergence to local minima, the algorithm needed to be started with a grid of pseudorandom starting points generated within two bounds (multistart technique). So, each fit procedure was done two-hundred-fold, with two-hundred different initializations. For each fit procedure, delays were determined as the time between the beginning of tissular enhancement and the beginning of arterial enhancement in celiac trunk. These starting points are chosen as the maximum of second-order derivative of tissue time activity curve and arterial input function. From this step, three perfusion parametric maps were obtained, one for each parameter of the model used.

### 2.5. Distributed Processing

The processing step was parallelized and executed on EGI within the biomed virtual organization (VO). The parallelization was handled at the input data level, by splitting each volume into several pieces. Each piece was processed by independent jobs running in parallel on multiple grid resources and eventually merged. The whole processing operation was modeled and implemented as a grid workflow using the Gwendia language [17] and the MOTEUR workflow engine [18]. The splitting and merging algorithms were developed in C++, while the processing algorithm was developed in Matlab. All three programs were compiled on a grid compliant operating system (CentOS) and deployed on the fly on the grid nodes. For the Matlab code we used the Matlab Compiler and the Matlab Compiler Runtime (MCR).

The interface with the grid resources was provided by the VIP web platform (https://vip.creatis.insa-lyon.fr/) [19]. A specific cartography workflow was developed for this application and integrated into the VIP platform.

The user uploaded the input volumes on the grid and launched the processing workflow from a web portal. In order to evaluate the speedup provided by our parallel approach, the total CPU time to make span ratio was determined. The makespan was defined as the time elapsed between the launch and the completion of the workflow, and the total CPU time as the sum of CPU times of all jobs in a workflow.

### 2.6. Statistical Analysis

In order to evaluate the reproducibility of our distributed computing algorithm, the 3D mapping procedures (workflow) were repeated three times for each subject. Relative standard variation (coefficient of variations) was then mapped for each parametric map for all patients and defined as the standard deviation to arithmetic mean ratio.

Next, to evaluate the accuracy of our method, results between ROI-based quantification method described in [12] and the method presented in this paper were compared. Quantitative perfusion parameters from three ROIs were calculated and averaged. The difference between methods was evaluated using the Bland-Altman representation for each perfusion parameters, the Spearman's coefficient calculation, and the nonparametric Wilcoxon test.

## 3. Results

### 3.1. Subjects

Among the 6 biopsied patients, histological results were as follows: 2 patients were scored *F*0, 3 patients scored *F*2, and 1 patient scored *F*4. 

### 3.2. Quantification Results

**Figure 1:**
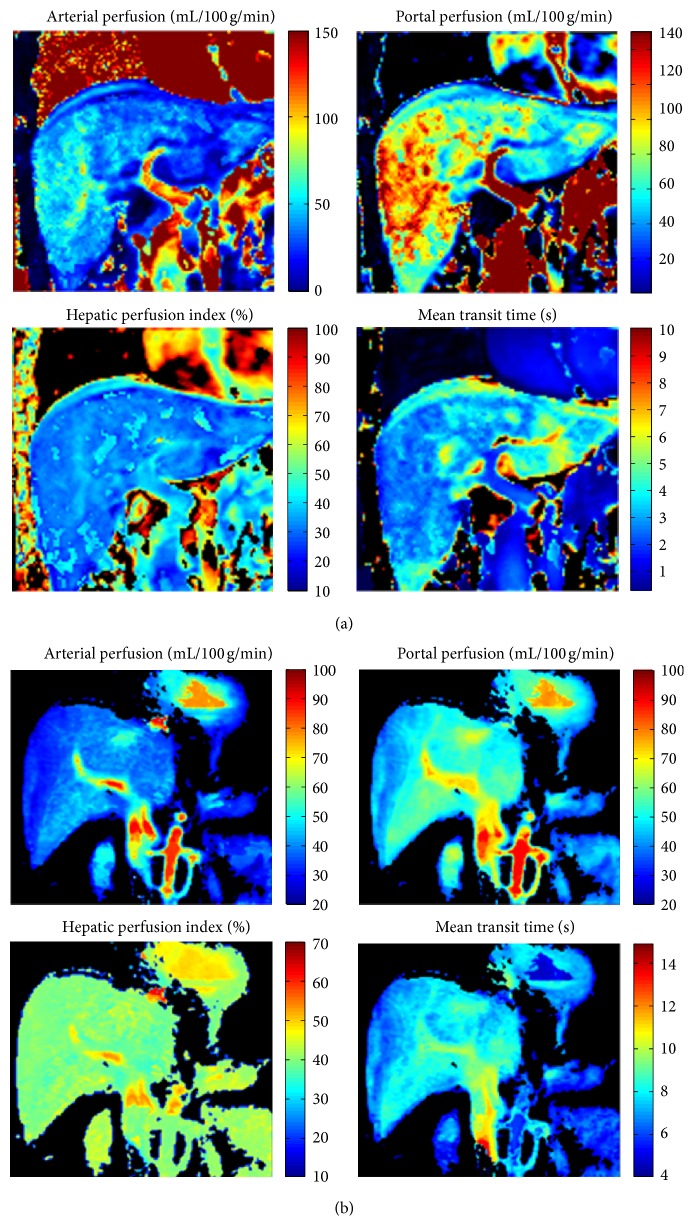
Representative liver perfusion parametric maps: arterial perfusion, portal perfusion, mean transit Time (MTT), and Hepatic perfusion Index (HPI) computed on a healthy subject (a) and on a patient with chronic liver diseases classified *F*2 according to METAVIR classification (b).

A representative set of 2D parametric maps extracted from 3D volumes on the healthy patient (METAVIR *F*0) is shown in Figure 1. Perfusion parameters values computed from the two compared methods are summarized in Table 1. Then, parameter values were stratified according to the fibrosis severity. Results corresponding to the advanced stage *(METAVIR stage *≥* F2)* and early stage* (METAVIR stage *<* F2)* are presented in Table 2.

### 3.3. Statistical Analysis

A significant correlation was observed between ROI-based method and distributed method for each parameter. Spearman's coefficients (*ρ*) were 0.86, 0.92, and 0.80 (*P* < 0.01) for arterial perfusion, portal perfusion and MTT, respectively. Regarding the Wilcoxon test and the Bland-Altman representations (Figure 3), significant difference was shown between compared methods. However, Bland-Altman representations showed a systematic decrease of MTTs values calculated with distributed method compared to ROI-based reference method.

About method reproducibility, all computed relative standard variation maps were null or negligible.

### 3.4. Distributed Processing Performance

**Figure 2:**
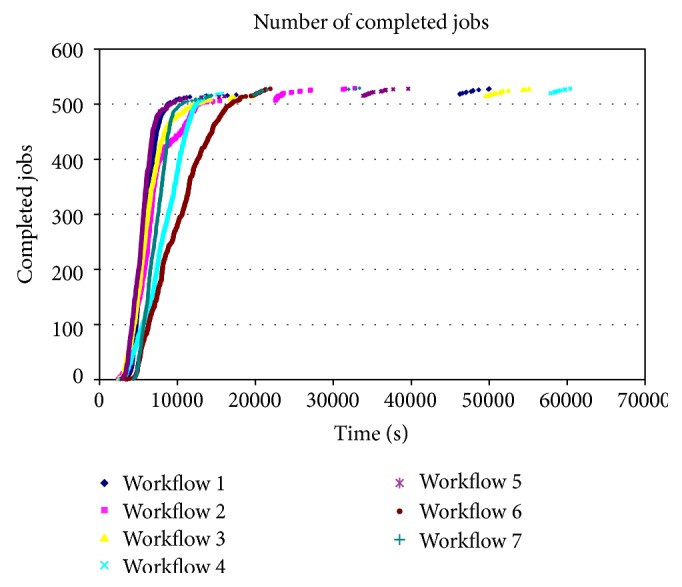
Number of completed jobs over time. Each workflow needs to wait for the completion of all its jobs in order to produce the final result. While most of the jobs finish within 10000 seconds, the last ones need much more time to complete. These last jobs almost triple the makespan (from roughly five to fifteen hours).

**Figure 3:**
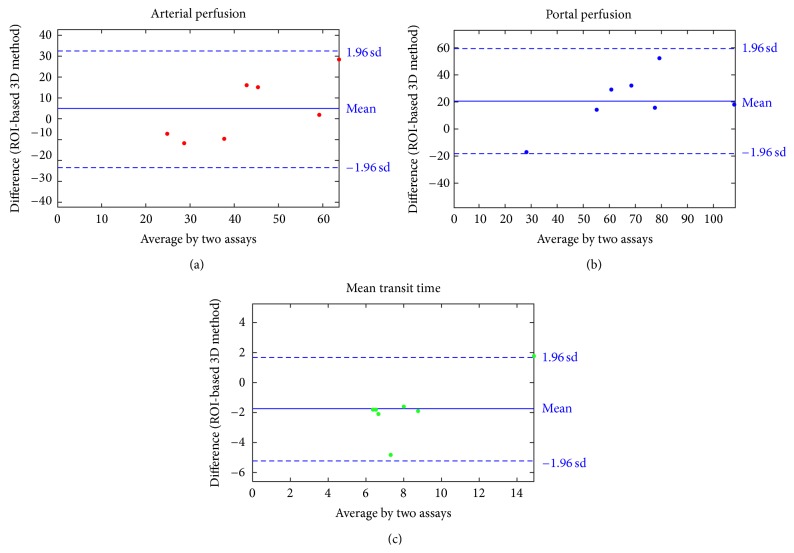
Bland-Altman representations computed for each perfusion parameters: (a) arterial perfusion, (b) portal perfusion, and (c) mean transit time quantified with the ROI-based method and the distributed method.

The major drawback of the perfusion-based method, its prohibitive computing time, has been overcome with the help of the EGI. By using the resources of distributed European infrastructure, 1 CPU year (corresponding to twenty-one 3D mapping procedures) was computed in only 9.5 days. The speedup varied among the 21 workflows from 20 to 94 with an average value of 48. The average error ratio for the experiments presented here was of 18%, with a maximum of 43% for one of the workflows. As shown in Figure 2, the late completion of the last jobs significantly increases the makespan. Most of the jobs finish within 10000 seconds. Nevertheless, a small percentage of the jobs need much more time to complete. These are typically failed jobs that need to be resubmitted one or multiple times. The workflow needs to wait for the completion of all jobs in order to produce the final result. Thus, for the experiments presented here, the makespan was almost tripled (from roughly five to fifteen hours) because of these late jobs. Similar results have been reported in other studies such as [20], where the authors propose a dynamic load balancing approach in order to improve performance. Nevertheless, the dynamic load balancing approach proposed in [20] only works for Monte-Carlo-based simulations.

## 4. Discussion

The presented method was reproducible and results were correlated with ROI-based reference method run locally on a personal computer. The quantified parameters were found to be in the same range as those obtained with the reference ROI-based method and those related in the literature[9, 10, 12]. Nevertheless, patient-wise, quantified values were slightly modified even if the shift was not found significant. Indeed, compared to the ROI-based method, blood flow quantified with the presented 3D method is overestimated whereas; on the contrary, MTTs are underestimated. Additionally, for each parameter, standard deviation observed with 3D methods run on EGI was found lower compared to the ROI-based method. When results are globally stratified according to fibrosis severity, the difference between mean values for each parameter computed with presented method is systematically lower than with ROI-based method. These findings confirm the smoothing effect induced by 3D quantification algorithm. Indeed, in ROI-based estimation method, arterial and portal delays are optimally set by user. However, these delays depend on spatial location and take into account the time shift between the measured input functions and the position where modeling takes place in parenchyma. Hence, manual setting is not possible in the 3D case, and an automatic estimate of both delays was mandatory. Due to relatively low image signal-to-noise ratio (SNR) of about eighteen, this step requires hard smoothing filtering, affecting quantification results with the acquisition data currently available. Another limitation is the restricted exploration volume. Indeed, to keep an acceptable SNR acquired with a high temporal resolution of 1 sec, the number of encoding steps in the slice-encoding direction was limited and the whole liver volume was not covered. These restrictions (SNR and coverage) can be overcome with the latest imaging MR systems with improved acquisition capabilities using 32 receiver channels with multiple element array coils. An SNR value of 80 was measured based on preliminary test performed at our institution with a 3T GEHC MR 750 (GEHC, Milwaukee, WI, USA) with 32 ch body coil. The parallelization of the method brings significant speedup and renders it feasible despite its prohibitive computing time. Nevertheless, performance can be still significantly improved. Currently the poor scheduling of the last tasks is largely due to platform heterogeneity and multiple task resubmissions caused by high error ratios. Data transfers account for most of the errors, while the rest are mostly application failures due to improper grid node configuration. 

As future work, scheduling will be improved by taking into account these considerations. 

To conclude, this preliminary study demonstrated that the described method allows 3D liver perfusion quantification within a reasonable processing time. It is now suitable to be used for similar clinical studies in a research context. While the distributed processing method was validated compared to the ROI-based quantification, such fully automatic processing requires high-quality images. The required SNR, together with a high temporal resolution and large volume exploration, can now be achieved on the latest 3T MRI systems available. Further work will have to demonstrate the interest of parametric 3D perfusion maps for fibrosis assessment on a larger number of subjects with chronic liver disease.

## Figures and Tables

**Table 1:** Quantified mean values of perfusion parameters for all subject obtained with the two compared methods.

Method	Arterial perfusion (mL·min^−1^·100 g^−1^)	Portal perfusion (mL·min^−1^·100 g^−1^)	MTT (s)
ROI-based (reference)	32.9 ± 20.8	78.7 ± 31.7	7.5 ± 3.8
3D (presented)	40.7 ± 10.2	57.9 ± 20.6	8.9 ± 1.6

**Table 2:** Mean values of perfusion parameters stratified according to fibrosis severity (advanced, METAVIR stage ≥ *F*2 and not advanced, METAVIR stage < *F*2) obtained with the presented method.

Fibrosis stage		Arterial perfusion (mL·min^−1^·100 g^−1^)	Portal Perfusion (mL·min^−1^·100 g^−1^)	MTT (s)
stage < *F*2	3D	35.0 ± 7.1	72.9 ± 26.3	8.7 ± 1.2
	Ref	25.6 ± 6.3	92.6 ± 26.3	6.9 ± 1.1
stage ≥ *F*2	3D	44.9 ± 10.9	47.6 ± 7.5	9.1 ± 2.1
	Ref	60.4 ± 12.3	68.2 ± 7.5	8.0 ± 5.2
